# Oxidized High-Density Lipoprotein Shows a Stepwise Increase as Fibrosis Progresses in Patients with Nonalcoholic Fatty Liver Disease

**DOI:** 10.3390/antiox10020239

**Published:** 2021-02-04

**Authors:** Kouichi Miura, Naoshi Arai, Rie Goka, Naoki Morimoto, Shunji Watanabe, Norio Isoda, Hironori Yamamoto, Kazuhiko Kotani

**Affiliations:** 1 Department of Medicine, Division of Gastroenterology, Jichi Medical University, Shimotsuke, Tochigi 329-0498, Japan; n.arai@jichi.ac.jp (N.A.); m04027rg@jichi.ac.jp (R.G.); morimoto@jichi.ac.jp (N.M.); 95103sw@jichi.ac.jp (S.W.); isodano1@jichi.ac.jp (N.I.); ireef@jichi.ac.jp (H.Y.); 2 Center for Community Medicine, Division of Community and Family Medicine, Jichi Medical University, Shimotsuke, Tochigi 329-0498, Japan; kazukotani@jichi.ac.jp

**Keywords:** NAFLD, liver fibrosis, coronary heart disease, oxidized HDL

## Abstract

Patients with nonalcoholic fatty liver disease (NAFLD) show dyslipidemia and a high risk for coronary heart disease (CHD). However, conventional atherosclerotic lipids are found at low levels in NAFLD patients with advanced fibrosis, in whom the risk for CHD is extremely high. The aim of the present study was to evaluate the levels of oxidized high-density lipoprotein (oxHDL), an emerging atherosclerotic biomarker, in patients with NAFLD. A total of 32 non-NAFLD subjects and 106 patients with NAFLD were enrolled. The fibrosis grades were stratified using non-invasive methods, including the Fibrosis-4 index and NAFLD fibrosis score. Total cholesterol and low-density lipoprotein (LDL)-cholesterol levels were significantly low in patients with advanced liver fibrosis. In contrast, oxHDL levels were high in NAFLD patients and showed a stepwise increase as fibrosis progressed. These oxHDL levels were independent of the HDL cholesterol levels, and statin use did not influence the oxHDL levels. Obese patients showed no increase in oxHDL levels, whereas patients with a low handgrip strength showed high oxHDL levels in NAFLD with advanced fibrosis. In conclusion, oxHDL is a potential biomarker for assessing the status of patients with NAFLD, including CHD and metabolic/nutritional disturbance, and particular cases with advanced liver fibrosis.

## 1. Introduction

Nonalcoholic fatty liver disease (NAFLD) is a hepatic feature of metabolic syndrome. Sedative lifestyles and high caloric diets, in combination with genetic background, contribute to the development of NAFLD [1]. The number of patients with NAFLD is sharply increasing worldwide. NAFLD and nonalcoholic steatohepatitis (NASH) can progress to cirrhosis and liver cancer. In addition, the leading cause of death in patients with NAFLD is cardiovascular diseases (CVDs) [2]. Thus, we must pay close attention to CVDs as well as hepatic complications in patients with NAFLD. Patients with NAFLD are characterized by dyslipidemia, including hypercholesterolemia, hypertriglyceridemia, and low concentrations of high-density lipoprotein (HDL) cholesterol [3]. Among lipid species, cholesterol plays an important role in the development of NAFLD in experimental models [4] as well as clinical settings [5]. Indeed, cholesterol-lowering agents can decrease transaminases in patients with NAFLD [6].

Although hypercholesterolemia and hypertriglycemia are well-known features of NAFLD, patients with NASH cirrhosis do not always show high levels of conventional atherogenic lipids, including cholesterol and triglyceride, due to impaired lipid synthesis in cirrhosis. As a result, the risk of CVDs may be underestimated in patients with advanced liver fibrosis. Even in low levels of conventional atherogenic lipids, NAFLD patients with advanced liver fibrosis showed increased morbidity [7] and mortality [8] of CVDs. Indeed, the guideline of the American Association for the Study of Liver Disease recommends that NASH cirrhosis is a screening target for CVDs [9]. However, an efficient screening for CVDs at follow-up has not been established. 

To resolve the issue in seeing patients with NAFLD, we focused on oxidized HDL (oxHDL), an emerging atherogenic biomarker. HDL cholesterol is recognized as a “good lipid” because HDL can remove cholesterol from macrophages in atherosclerotic lesions and subsequently deliver it to the liver [10]. Indeed, low levels of HDL cholesterol are reported in patients with CVDs. However, elevation of HDL cholesterol levels does not always improve the mortality of CVD patients. The quality of HDL is another aspect that accounts for the paradox between HDL cholesterol levels and CVDs. Modified HDL can attenuate the native functions of HDL, including macrophage cholesterol efflux, anti-atherogenic properties, and antioxidative activity. Importantly, oxHDL has opposite functions to naïve HDL, and oxHDL levels are actually elevated in patients with CVDs [11,12].

At present, little information on oxHDL is available in patients with NAFLD. We therefore investigated the oxHDL levels in patients with NAFLD. Our data provide new information on the follow-up system for patients with NAFLD.

## 2. Methods

### 2.1. Patients

Non-NAFLD subjects and patients with NAFLD were recruited at Hotaka Hospital and Jichi Medical University Hospital, respectively. The diagnosis of NAFLD was made based on the presence of hepatic steatosis and exclusion of other liver diseases. In all subjects and patients with NAFLD, alcohol use was <30 g/day and <20 g/day for men and women, respectively. Chronic hepatitis C, hepatitis B virus infection, autoimmune hepatitis, primary biliary cholangitis, and other liver diseases were excluded by serological examinations. Steatosis was determined by an ultrasonographic examination conducted by well-experienced gastroenterologists.

The present study was approved by the Institutional Review Board of Jichi Medical University (permission no. CL17-M158, A18-102) and conducted according to the Declaration of Helsinki. Written informed consent was obtained from all participants who enrolled in the present study.

### 2.2. Collection of Clinical Data

Blood counts and chemistry were examined with automated analyzers. Among several biomarkers that can avoid liver biopsy in an assessment of liver fibrosis [13], we used a non-invasive scoring system, including the Fibrosis-4 (FIB-4) index and NFS. The FIB-4 index is a universal scoring system, and the NAFLD fibrosis score (NFS) is specific for NAFLD; these values were calculated according to the published formulae [14,15]. Using these formulae, patients with NAFLD were divided into three groups (Table 1): group 1, low probability of fibrosis; group 2, intermediate probability of fibrosis; and group 3, high probability of advanced liver fibrosis corresponding to stage 3 and 4 [16]. Because FIB-4 and NFS were independently used for the grouping, the number of patients in a categorized group differ between FIB-4 and NFS (i.e., group 1 in FIB-4 and group 1 in NFS).

The characteristic variables were collected based on the clinical records as follows: the current smoking status, body mass index (BMI; calculated as weight (kg) divided by height (m) squared), hypertension (defined as a systolic blood pressure ≥ 140 mmHg, diastolic blood pressure ≥ 90 mmHg, and/or anti-hypertensive drug use), diabetes mellitus (defined as a fasting blood glucose ≥ 126 mg/dL, hemoglobin A1c (HbA1c) ≥ 6.5% and/or anti-diabetic drug use), dyslipidemia (defined as low-density lipoprotein (LDL) cholesterol ≥ 140 mg/dL, triglyceride ≥ 150 mg/dL and/or use of lipid-lowering drugs), laboratory data related to liver conditions (i.e., aspartate aminotransferase (AST), alanine aminotransferase (ALT), γ-GTP, albumin), and laboratory data of lipids and cholesterol-lowering drug use (i.e., statins). We also measured muscle weight, fat weight, and % of body fat mass by bioimpedance analysis (InBody770^®^, InBody Japan, Tokyo, Japan). The oxHDL levels of serum from each subject were measured by an enzyme-linked immune-sorbent assay (ELISA), as previously reported [17]. Serum oxLDL was measured using an ELISA kit (Mercodia, Uppsala, Sweden) according to the manufacturer’s instruction.

### 2.3. The Assessment of Handgrip Strength

Handgrip strength was measured using the Smedley’s hand dynamometer according to the manufacturer’s instructions. In brief, the test was performed with both right and left hands in a standing position, unless the hand was injured or involved in muscle/neuro-related diseases. The average of the two hands was used. Because handgrip strength depends on age, we defined “low strength” when the measurement was lower than that of the Japanese average after stratification by age, which was determined by the Japan Sports Agency (https://www.mext.go.jp/sports/b_menu/toukei/chousa04/tairyoku/kekka/k_detail/1421920.htm).

### 2.4. Statistical Analyses

Categorical variables were expressed as the number (percentage), and continuous variables were expressed as the means ± standard deviation or median (interquartile range) according to the distribution. The differences between the two groups were examined using the chi-square test, Fisher’s exact test, Student’s *t*-test, or Mann–Whitney U-test analysis as appropriate. Statistical analyses were performed with the EZR software program (version 1.51; Jichi Medical University Saitama Medical Center, Saitama, Japan), which is a graphical user interface for R (version 3.6.3; R Foundation for Statistical Computing, Vienna, Austria). It is a modified version of R Commander designed to add statistical functions used in biostatistics [18]. Graphs were created using the STATA software program, version 15.1 (Stata Corp, College Station, TX, USA). The differences among three or four groups were examined using a one-way analysis of variance (ANOVA) with Tukey’s multi-comparison test or the Kruskal–Wallis test with the Steel–Dwass multi-comparison test for continuous variables, and Fisher’s exact test with Holm’s correction for categorical variables. A value of *p* < 0.05 was considered to indicate significance.

## 3. Results

### 3.1. Characterization of Patients with NAFLD

A total of 32 non-NAFLD subjects and 106 patients with NAFLD were enrolled in the present study. Table 2 shows the characteristics of the subjects. Patients with NAFLD had a high prevalence of diabetes mellitus, dyslipidemia, and hypertension, with 28.3%, 36.8%, and 45.3% of these patients, respectively, taking medications for the corresponding disease. The NAFLD group had an older age, higher BMI, and higher transaminases levels than the non-NAFLD group (Table 2). In addition, the NAFLD group showed higher scores for the FIB-4 index and NFS than the non-NAFLD group. The NAFLD group included 24 patients with liver cirrhosis, as determined by imaging studies (computed tomography and/or ultrasonography).

### 3.2. Low Conventional Atherosclerotic Lipids Levels in Patients with Advanced Liver Fibrosis

**Figure 1:**
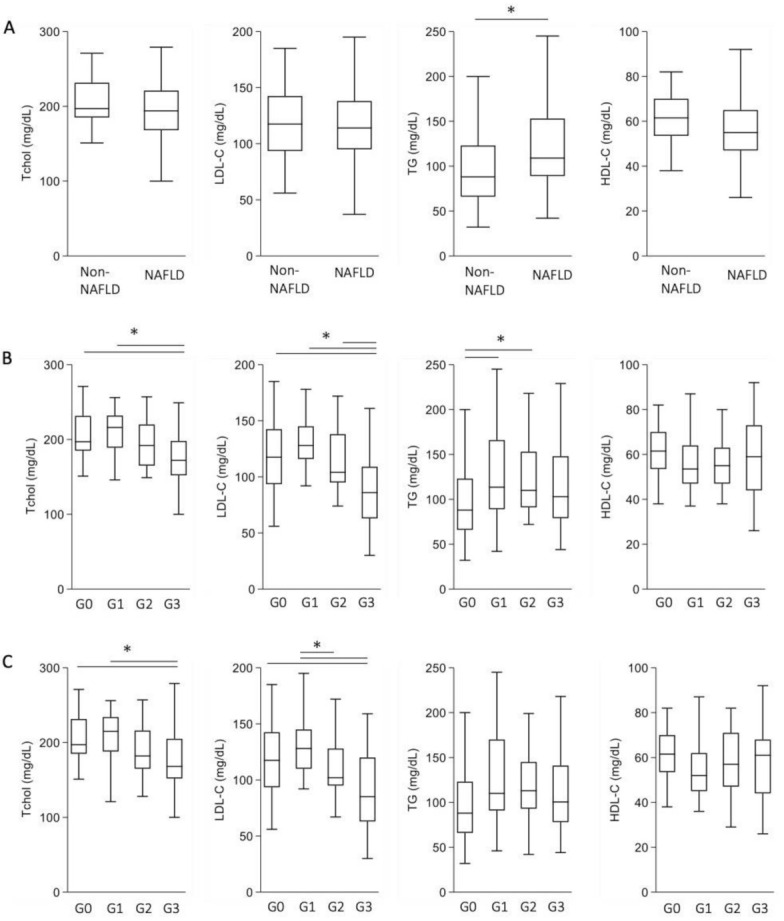
The levels of total cholesterol, low-density lipoprotein (LDL) cholesterol, triglyceride, and high-density lipoprotein (HDL) cholesterol in non-NAFLD subjects and patients with NAFLD. (**A**) Non-NAFLD (*n* = 32) versus NAFLD (*n* = 106). (**B**). Lipid levels after stratification by the FIB-4 index. G0, Non-NAFLD (*n* = 32); G1, group 1 (*n* = 38); G2, group 2 (*n* = 35); G3, group 3 (*n* = 33). (**C**) Lipid levels after stratification by the NFS. G0, Non-NAFLD (*n* = 32); G1, group 1 (*n* = 47); G2, group 2 (*n* = 33); G3, group 3 (*n* = 26). * *p* < 0.05.

We investigated the levels of conventional atherosclerotic lipids, including total cholesterol, LDL cholesterol, and triglyceride. There were no marked differences in the levels of total cholesterol and LDL cholesterol between the non-NAFLD and NAFLD groups (Figure 1A). The triglyceride levels were higher in the NAFLD group than in the non-NAFLD group. In contrast, the levels of HDL cholesterol, an anti-atherosclerotic lipid, tended to be lower in the NAFLD group than in the non-NAFLD group (Figure 1A).

We then investigated the levels of these lipids in each fibrosis grade: group 1, low probability of fibrosis; group 2, intermediate probability of fibrosis; and group 3, high probability of advanced fibrosis. The background characteristics of each group are provided as Supplemental Materials (Supplementary Tables S1 and S2). As expected, group 3 in FIB-4 stratification showed a high NFS, and group 3 in NFS stratification showed a high FIB-4 index. The prevalence of liver cirrhosis was high in group 3 for both stratifications, but no patients in group 1 of either stratification showed liver cirrhosis. In addition, the prevalence of diabetes showed a stepwise increase as the fibrosis score increased. In the FIB-4 index stratification (Figure 1B), the total cholesterol and LDL cholesterol levels tended to be high in group 1 but were significantly low in group 3. The triglyceride levels were high in groups 1 and 2 but not in group 3. The HDL cholesterol levels were not significantly different for any stratification of fibrosis. The use of lipid-lowering agents was similar among all fibrosis groups. NFS stratification also showed low levels of total cholesterol and LDL cholesterol in group 3 (Figure 1C). Thus, the levels of total cholesterol and LDL cholesterol were low in group 3 compared with the non-NAFLD group.

### 3.3. Stepwise Increase in oxHDL as Liver Fibrosis Progresses

**Figure 2:**
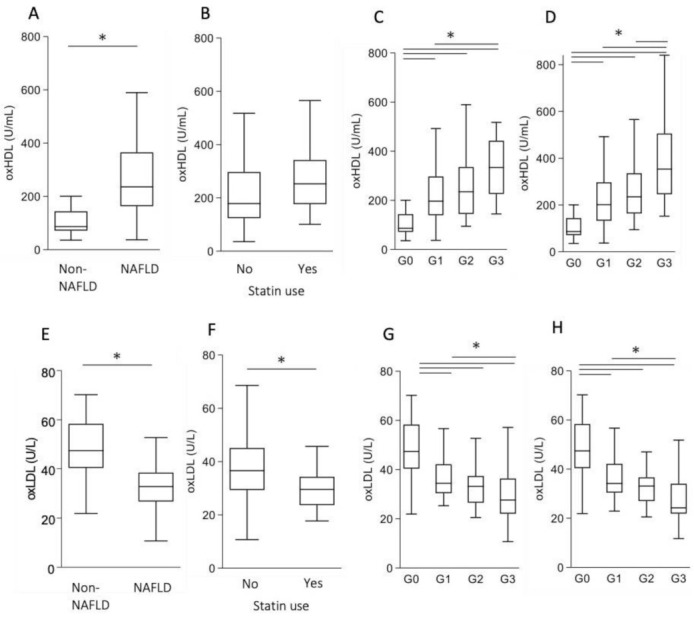
The levels of oxidized HDL (oxHDL) and oxLDL in non-NAFLD subjects and patients with NAFLD. (**A**–**D**) oxHDL levels. (**E**–**H**) oxLDL levels. (**A**,**E**) Non-NAFLD (*n* = 32) versus NAFLD (*n* = 106). (**B**,**F**) Non-statin use (*n* = 77) versus statin use (*n* = 29). (**C**,**G**) Lipid levels after stratification by the FIB-4 index. (**D**,**H**) Lipid levels after stratification by the NFS. * *p* < 0.05.

Although the prevalence of coronary heart disease (CHD) is reported to be high in patients with advanced fibrosis [7], atherosclerotic lipid levels were low in group 3, an advanced liver fibrosis group. Thus, we examined the serum levels of modified lipoproteins, including oxHDL and oxLDL. The median values of oxHDL in the non-NAFLD and NAFLD groups were 86.5 U/mL and 235.6 U/mL, respectively, being significantly higher in the NAFLD group (Figure 2A). Statin use did not influence the oxHDL levels (Figure 2B). In the FIB-4 stratification, oxHDL displayed a stepwise increase as fibrosis progressed (Figure 2C), with a similar trend also noted in the NFS stratification (Figure 2D).

In contrast, oxLDL levels were significantly lower in the NAFLD group than in the non-NAFLD group. The median oxLDL levels in the non-NAFLD and NAFLD groups were 47.4 U/L and 32.8 U/L, respectively (Figure 2E). Statin use significantly decreased oxLDL levels (Figure 2F). On stratifications using the FIB-4 index and NFS, oxLDL showed a stepwise decrease as fibrosis progressed (Figure 2G,H).

### 3.4. Association of a Low Handgrip Strength with Increased oxHDL Levels in Patients with Advanced Fibrosis

**Figure 3:**
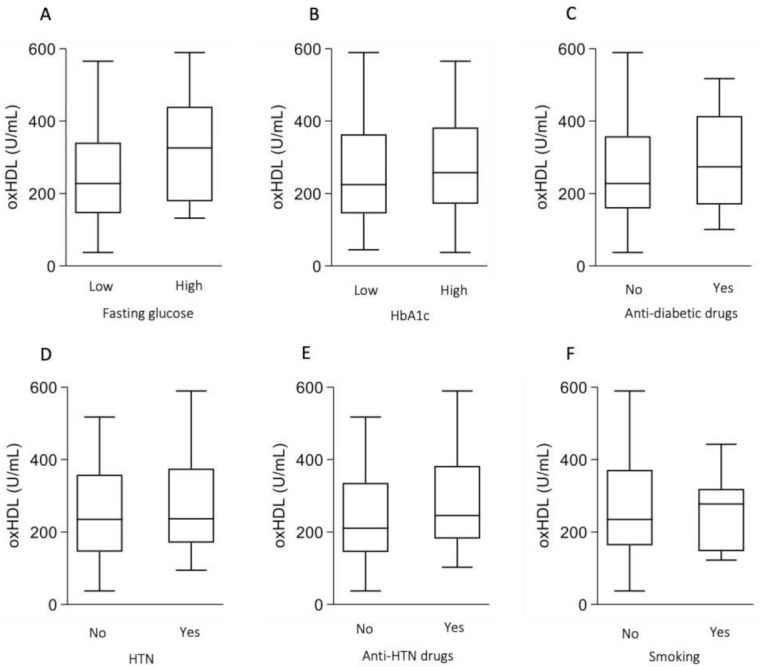
oxHDL levels in each group among patients with NAFLD. (**A**) Low fasting glucose (<126 mg/dL, *n* = 79) versus high fasting glucose concentration (≥126 mg/dL, *n* = 27). (**B**) Low HbA1c (<6.5%, *n* = 71) versus high HbA1c (≥6.5%, *n* = 33). (**C**) Non-use (*n* = 43) versus use of anti-diabetic drugs (*n* = 63). (**D**) No hypertension (HTN) (*n* = 43) versus hypertension (HTN) (*n* = 63). (**E**) Non-use (*n* = 57) and use of anti-HTN drugs (*n* = 49). (**F**) Non-smoker (*n* = 98) versus smoker (*n* = 8).

**Figure 4:**
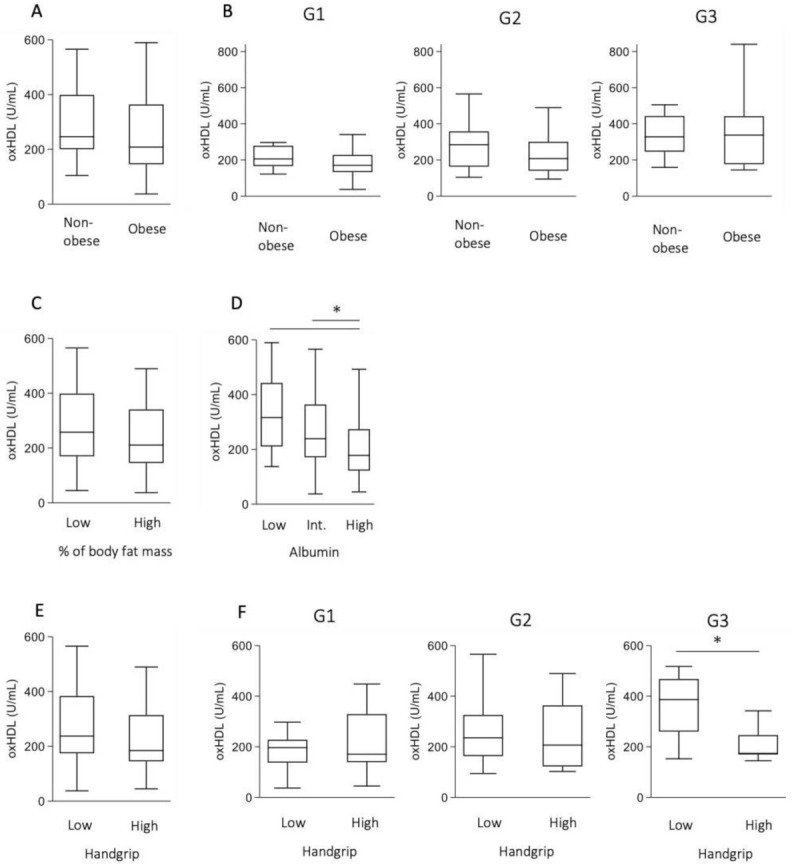
oxHDL levels in patients with NAFLD. (**A**) Non-obese (*n* = 33) versus obese individuals (*n* = 73). (**B**) Non-obese versus obese individuals in each fibrosis grade defined by the FIB-4 index. G1, Non-obese (*n* = 19) versus obese individuals (*n* = 27); G2, Non-obese (*n* = 7) versus obese individuals (*n* = 30); G3, Non-obese (*n* = 9) versus obese individuals (*n* = 16). (**C**) Individuals with low (*n* = 37) versus high percentage of body fat mass (*n* = 67). High percentage of body fat mass was defined >30% in men and >35% in women. (**D**) Low (*n* = 26) versus intermediate (*n* = 51) versus high albumin group (*n* = 29). Albumin concentrations in each group were <4.1, 4.1–4.5, <4.5 g/dL, respectively. (**E**). Low (*n* = 72) versus high handgrip strength (*n* = 28). (**F**) Low versus high handgrip strength in each fibrosis grade defined by the FIB-4 index. G1, low (*n* = 27) versus high (*n* = 16); G2, low (*n* = 26) versus high (*n* = 8); G3 low (*n* = 19) versus high (*n* = 4). * *p* < 0.05.

Because patients with NAFLD frequently have metabolic and nutritional disturbances, we examined the association between oxHDL and the metabolic/nutrition status. Although oxHDL levels tended to be elevated in high-glucose and high-HbA1c populations among patients with NAFLD, these findings were not statistically significant (Figure 3A,B). Anti-diabetic drugs did not change the oxHDL levels (Figure 3C). In addition, the presence of hypertension and the use of anti-hypertensive drugs did not change the oxHDL levels (Figure 3D,E). Current smoking status did not increase the oxHDL levels (Figure 3F). Because obesity is a risk factor for CHD, we speculated that obesity might increase the oxHDL levels. However, the presence of obesity, set as a BMI > 25, did not increase the oxHDL levels (Figure 4A). This trend was noted even after stratification of fibrosis (Figure 4B). In a comparison of percent body fat mass, no significant difference was observed between low and high groups (Figure 4C). On the other hand, the levels of oxHDL decreased as albumin levels increased (Figure 4D). We therefore focused on non-obese patients with NAFLD who might have sarcopenia. Because the number of patients with sarcopenia was small in the present study, we examined the association between handgrip strength and oxHDL. Among patients with NAFLD, the oxHDL levels tended to be high in the low-handgrip-strength population (Figure 4E). We then examined the association between the oxHDL levels and handgrip strength in each fibrosis grade. Although the oxHDL levels were not significantly different between low- and high-handgrip-strength populations in groups 1 and 2, the low-handgrip-strength population showed significantly high oxHDL levels in group 3 (Figure 4F).

## 4. Discussion

In the present study, we showed that the oxHDL levels were higher in the NAFLD group than in the non-NAFLD group. In addition, the oxHDL levels showed a stepwise increase as liver fibrosis progressed. Furthermore, a low handgrip strength but not obesity was associated with high oxHDL levels in patients with advanced liver fibrosis. Thus, oxHDL is a potential biomarker for assessing the status of patients with NAFLD.

oxHDL has the potential to assess the risk of CHD when conventional atherosclerotic lipid levels are low in patients with advanced liver fibrosis. Indeed, oxHDL levels were reported to be high in patients with CVDs [11,12]. At present, there are few markers that broadly cover the risk assessment of CHD in patients with NAFLD. Although conventional atherosclerotic lipids can be used for risk assessment of CHD in patients with less fibrosis (group 1 in the present study), these lipid levels were low in patients with advanced fibrosis (group 3 in the present study). HDL cholesterol can be used as a marker for assessing the risk for CHD because the HDL cholesterol levels were found to be low in patients with cirrhosis [19]. However, HDL cholesterol levels were not decreased in the advanced fibrosis group (group 3) in the present study. A low HDL cholesterol level may represent the terminal stage of liver function. As a result, the risk for CHD may be underestimated in patients with advanced fibrosis when the levels of conventional atherosclerotic lipids are used to assess the risk of CHD. Because the liver is the major organ that synthesizes cholesterol, patients with cirrhosis show decreased cholesterol synthesis [20]. In contrast, oxHDL was not associated with conventional atherosclerotic lipids, suggesting that the capacity of cholesterol synthesis had little effect on the oxHDL levels. Thus, oxHDL may be useful as an alternative biomarker that broadly covers the risk assessment of CHD in patients with NAFLD.

oxHDL can be used to assess the risk of CHD when patients receive lipid-lowering drugs, including statins. Although lipid-lowering drugs aim to reduce serum levels of conventional atherosclerotic lipids, they may mask the risk of CHD in patients with NAFLD. In the present study, 36.8% of patients with NAFLD had already received lipid-lowering drugs when they visited our hospitals for further evaluation of their liver dysfunction. As a result, the total cholesterol and LDL cholesterol levels were similar between the control and NAFLD groups. In addition, these atherosclerotic lipid levels were low in patients with advanced liver fibrosis, with the use of lipid-lowering drugs calculated to be 38.1% in group 3 according to FIB-4 stratification. In contrast, statin use had little effect on the oxHDL levels. We also measured the levels of oxLDL, which has potent proinflammatory activity and can damage vascular endothelial cells [21]. Although oxLDL levels were reported to be high in patients with NAFLD [22] and CVDs [23], the levels were significantly low in patients with advanced fibrosis in the present study. In addition, statin use significantly decreased the oxLDL levels. Thus, oxHDL helps to clarify the underlying risk of CHD even when patients receive lipid-lowering drugs.

oxHDL has the potential to assess the metabolic/nutritional disturbance in patients with NAFLD. The association between metabolic/nutritional disturbance and oxHDL levels is of interest. We initially speculated that obesity and diabetes would increase the oxHDL levels. However, patients with obesity showed no increase in oxHDL. In addition, the presence of diabetes had only slight effects on the oxHDL levels in patients with NAFLD. These findings prompted us to examine the association between the oxHDL levels and handgrip strength, which has recently received focus as another aspect of metabolic/nutritional disturbance. Although the number of subjects was small, patients with a low handgrip strength showed high oxHDL levels in the advanced fibrosis groups. Currently, low handgrip strength is a risk factor for NAFLD [24]. Sarcopenia is associated with NAFLD, independent of obesity [25]. Recently, a high prevalence of CVDs has been reported in patients with a low handgrip strength [26]. These findings may link those of a previous report showing the risk of CVDs in non-overweight NAFLD patients was higher than that in overweight NAFLD patients [27].

oxHDL is a useful biomarker among multiple forms of HDL, including HDL subclasses and dysfunctional HDL. HDL is heterogeneous, with some forms potentially being involved in the development of CVDs. HDL can be divided into several subclasses according to its size. Although these HDL subclasses may possess their own properties, the functions are likely to be disease-specific [10]. Because HDL subclasses have not obtained a consensus, it is difficult to handle HDL subclasses to assess the risk of CHD. Recently, the quality rather than the quantity of HDL has garnered attention concerning the development of atherosclerosis. HDL receives several modifications, including oxidation, glycation, and compositional changes in lipid contents [28]. These modifications of HDL lead to dysfunctional HDL, in which the native functions of HDL are reduced. Among dysfunctional HDLs, oxidative modification of HDL results in the loss of macrophage cholesterol efflux and the exertion of an atherogenic property. Because NAFLD is well known to generate massive oxidative stress, it makes sense that HDL sustains oxidative modification. As shown in the present study, the oxHDL levels were independent of the HDL cholesterol levels.

Although we demonstrated the potential utility of oxHDL in assessing the status of NAFLD, a couple of limitations associated with the present study warrant mention. First, our study did not have enough power to demonstrate the association between CHD and oxHDL in NAFLD due to the small number of patients enrolled. However, we showed for the first time the close association between oxHDL and liver fibrosis, which is a strong risk factor for CVDs in NAFLD patients. Second, we used non-invasive markers to evaluate liver fibrosis. Although a liver biopsy is still the gold standard for assessing liver fibrosis, non-invasive markers, including FIB-4 and NFS, are reliable for discriminating NASH cirrhosis without a liver biopsy [29]. In patients assigned to the advanced fibrosis group (group 3) based on these non-invasive methods, the prevalence of liver cirrhosis determined by imaging studies was extremely high. We therefore believe that most patients in group 3 did indeed have advanced liver fibrosis, corresponding to stage 3 to 4. In addition, there are several cut-off values of the FIB-4 index to predict advanced liver fibrosis. We also used another cut-off value of the FIB-4 index to predict advanced liver fibrosis for Japanese patients with NAFLD [30]. The results were almost similar to the presented data (data not shown). In addition, we also examined the association of oxHDL and Mac-2 binding protein glycan isomer, a marker for liver fibrosis, and reproduced similar results to the present data (data not shown). Thus, we considered that oxHDL increases as liver fibrosis progresses. Third, detailed mechanisms by which oxHDL increases in patients with NAFLD remain unknown. Food composition, physical activity, and insulin resistance are potential factors that may influence the levels of oxHDL. Further studies are required to clarify these issues.

In conclusion, we demonstrated that oxHDL is a potential biomarker for assessing the status of patients with NAFLD, including CVDs and metabolic/nutritional disturbance, particularly in patients with advanced fibrosis.

## Figures and Tables

**Table 1:** Stratification of liver fibrosis using non-invasive methods.

	Group 1	Group 2	Group 3
FIB-4	<1.30	1.30–2.67	>2.67
NFS	<−1.455	−1.455–0.675	>0.675

NFS: Nonalcoholic fatty liver disease (NAFLD) fibrosis score.

**Table 2:** Characteristics of subjects enrolled in the present study.

	Non-NAFLD	NAFLD	*p*-Value
*n*	32	106	
Male (%)	20 (62.5)	40 (37.7)	0.015
Diabetes mellitus (%)	0 (0.0)	47 (44.3)	<0.001
Dyslipidemia (%)	10 (31.3)	67 (63.2)	0.005
Hypertension (%)	6 (18.8)	63 (59.4)	<0.001
Obesity (%)	12 (37.5)	73 (68.9)	0.002
Smoking (%)	10 (31.3)	8 (7.5)	0.001
Liver cirrhosis (%)	0 (0.0)	24 (22.6)	<0.001
Age (year)	48.2 ± 7.9	58.3 ± 13.6	<0.001
BMI (kg/m^2^)	22.4 [20.4, 25.8]	27.2 [23.7, 30.5]	<0.001
Systolic BP (mmHg)	121 ± 19	132 ± 16	0.002
Diastolic BP (mmHg)	75 ± 15	77 ± 11	0.312
Platelet (× 10^4^/μL)	22.4 ± 4.4	20.3 ± 7.7	0.147
AST (U/L)	22 [18, 24]	37 [25, 50]	<0.001
ALT (U/L)	20 [15, 25]	39 ([29, 58]	<0.001
γ-GTP (U/L)	24 [17, 39]	46 [30, 81]	<0.001
Albumin (g/dL)	4.5 [4.3, 4.7]	4.3 [4.1, 4.5]	0.001
Total bilirubin (mg/dL)	0.90 [0.80, 1.10]	0.85 [0.64, 1.08]	0.234
Fasting glucose (mg/dL)	96 [93, 102]	106 [98, 126]	<0.001
HbA1c (%)	5.6 [5.4, 5.8]	6.1 [5.8, 6.8]	<0.001
FIB-4 index	1.05 [0.73, 1.28]	1.70 [0.96, 3.11]	<0.001
NFS	−2.35 [−2.94, −1.94]	−1.03 [−2.56, 0.63]	<0.001

NAFLD: nonalcoholic fatty liver disease; BMI: body mass index; BP: blood pressure; AST: aspartate aminotransferase; ALT: alanine aminotransferase; γ-GTP: γ-glutamyl transpeptidase, NFS: NAFLD fibrosis score. Continuous variables are mean ± standard deviation or median (interquartile range (Q1, Q3)).

## Data Availability

The data are presented within the paper. Additional raw data are available on request from the corresponding author.

## Supplementary Materials

The following are available online at https://www.mdpi.com/2076-3921/10/2/239/s1, Table S1. The characteristics of non-NAFLD subjects and patients with NAFLD after stratification of liver fibrosis using the FIB-4 index. Table S2. The characteristics of non-NAFLD subjects and patients with NAFLD after stratification of liver fibrosis using NFS.
