# Supplementary material for: Oxidized High-Density Lipoprotein Shows a Stepwise Increase as Fibrosis Progresses in Patients with Nonalcoholic Fatty Liver Disease
Source: Antioxidants (Basel). 2021 Feb 4;10(2):239. doi: 10.3390/antiox10020239 (PMC7914565; doi:10.3390/antiox10020239)
Supplement: Supplementary file 1 [file antioxidants-10-00239-s001.pdf]

Table S1. The characteristics of Non-NAFLD subjects and patients with NAFLD after stratification of liver fibrosis using FIB-4 index

Factor	Group 0	Group 1	Group 2	Group 3	P value
n	32	38	35	33	
Male (%)	20 (62.5) a	18 (47.4) a	12 (34.3) a	10 (30.3) a	0.039
Obesity (%)	12 (37.5) a	25 (67.6) a	24 (70.6) a	22 (66.7) a	0.023
Smoking (%)	10 (31.2) a	3 (7.9) a	3 (8.6) a	2 (6.1) a	0.016
DM (%)	2 (6.2) a	8 (21.1) a	19 (54.3) b	20 (60.6) b	<0.001
Anti DM drug (%)	0 (0.0) a	5 (13.2) ab	11 (31.4) bc	15 (45.5) c	<0.001
HTN (%)	6 (18.8) a	13 (34.2) a	27 (77.1) b	23 (69.7) b	<0.001
Anti HTN drug (%)	0 (0.0) a	7 (18.4) b	22 (62.9) c	20 (60.6) c	<0.001
HL (%)	10 (31.2) a	26 (68.4) b	23 (65.7) b	18 (54.5) ab	0.009
Statin use (%)	0 (0.0) a	6 (15.8) ab	13 (37.1) b	10 (30.3) b	<0.001
Liver cirrhosis (%)	0 (0.0) a	0 (0.0) b	3 (8.6) b	21 (63.6) c	<0.001
Low handgrip (%)	N/A a	22 (59.5) a	26 (78.8) a	24 (80.0) a	0.119
Age (year)	48.2±7.9 a	45.5±12.1 a	66.0±8.6 b	65.0±7.1 b	<0.001
sBP (mmHg)	121.2±18.6 a	125.7±14.8 a	141.1±15.3 b	130.4±15.3 ac	<0.001
dBP (mmHg)	74.9±14.8 a	77.3±10.3 a	80.8±10.7 a	74.0±10.6 a	0.1
Platelet (×10^4/μl)	22.4±4.4 a	26.7±5.4 b	21.0±4.7 ac	12.2±4.7 d	<0.001
BMI	22.4 [20.4, 25.8] a	27.3 [23.7, 30.9] b	27.1 [24.1, 30.9] b	26.8 [23.6, 29.7] b	<0.001
AST (U/l)	21.5 [17.8, 24.0] a	27.5 [23.3, 39.0] b	38.0 [27.0, 52.0] bc	44.0 [35.0, 61.0] c	<0.001
ALT (U/l)	20.0 [14.8, 25.0] a	41.0 [30.0, 66.0] b	41.0 [34.5, 63.0] b	32.0 [25.0, 48.0] b	<0.001
γ-GTP (U/l)	24.0 [16.5, 38.8] a	41.0 [27.8, 97.0] b	42.0 [31.0, 71.0] b	50.0 [33.0, 71.0] b	<0.001
Albumin (g/dl)	4.5 [4.3, 4.7] a	4.4 [4.2, 4.7] b	4.3 [4.2, 4.4] b	4.0 [3.6, 4.3] c	<0.001
TB (mg/dl)	0.9 [0.8, 1.1] a	0.8 [0.7, 1.0] a	0.9 [0.6, 1.0] a	1.00 [0.7, 1.9] a	0.236
FBS (mg/dl)	96 [93, 102] a	97 [94, 110] a	113 [103, 136] b	115 [100, 140] b	<0.001
HbA1c (%)	5.6 [5.4, 5.8] a	5.9 [5.6, 6.1] b	6.3 [6.0, 6.9] c	6.1 [5.9, 7.2] bc	<0.001
FIB-4 index	1.1 [0.7, 1.3] a	0.8 [0.6, 1.0] b	1.9 [1.5, 2.2] c	4.2 [3.3, 5.2] d	<0.001
NFS	-2.4 [-2.9, -1.9] a	-3.0 [-3.5, -2.2] a	-0.9 [-1.7, 0.4] b	1.2 [0.4, 1.8] c	<0.001

NAFLD, nonalcoholic fatty liver disease; DM, diabetes mellitus; HTN, hypertension; HL, hyperlipidemia; sBP, systolic blood pressure; dBP, diastolic blood pressure; BMI, body mass index; AST, aspartate aminotransferase; ALT, alanine aminotransferase; γ-GTP, γ-glutamyl transpeptidase; TB, Total bilirubin; FBS, fasting blood sugar; NFS, NAFLD fibrosis score. Continuous variables are mean ± standard deviation or median (interquartile range [Q1, Q3]). There are significant differences if different alphabets are labeled (i.e. a vs. b).

Table S2. The characteristics of Non-NAFLD subjects and patients with NAFLD after stratification of liver fibrosis using NFS.

Factor	Group 0	Group 1	Group 2	Group 3	P value
n	32	47	33	26	
Male (%)	20 (62.5) a	22 (46.8) a	11 (33.3) a	7 (26.9) a	0.025
Obesity (%)	12 (37.5) a	27 (60.0) ab	24 (72.7) b	20 (76.9) b	0.007
Smoking (%)	10 (31.2) a	4 (8.5) a	3 (9.1) a	1 (3.8) a	0.006
DM (%)	2 (6.2) a	9 (19.1) a	17 (51.5) b	21 (80.8) b	<0.001
Anti DM drug (%)	0 (0.0) a	7 (14.9) ab	9 (27.3) bc	15 (57.7) c	<0.001
HTN (%)	6 (18.8) a	18 (38.3) ab	27 (81.8) c	18 (69.2) bc	<0.001
Anti HTN drug (%)	0 (0.0) a	12 (25.5) b	22 (66.7) c	15 (57.7) c	<0.001
HL (%)	10 (31.2) a	34 (72.3) b	19 (57.6) ab	14 (53.8) ab	0.008
Statin use (%)	0 (0.0) a	11(23.4) b	9 (27.3) b	9 (34.6) b	0.004
Liver cirrhosis (%)	0 (0.0) a	0 (0.0) b	5 (15.2) c	19 (73.1) d	<0.001
Low handgrip (%)	N/A a	28 (60.9) b	25 (78.1) b	19 (73.1) b	N/A
Age (year)	48.2 ± 7.9 a	49.5 ± 13.7 a	64.2 ±7.6 b	66.9 ± 9.1 b	<0.001
sBP (mmHg)	121 ±19 a	128 ± 14 a	139 ± 17 b	131 ±16 ab	<0.001
dBP (mmHg)	75 ± 15a	78 ± 10 a	80 ±11 a	73 ±11 a	0.159
Platelet (×10^4/μl)	22.4 ± 4.4 a	26.2 ± 5.4 b	18.5 ± 5.2 c	11.8 ± 4.4 d	<0.001
BMI	22.4 [20.4, 25.8] a	26.70 [23.2, 29.6] b	27.8 [24.5, 30.9] b	27.8 [25.4, 30.2] b	<0.001
AST (U/l)	22 [18, 24] a	32 [24, 43] b	39 [29, 55] b	40 [29., 46] b	<0.001
ALT (U/l)	20 [15, 25] a	42 [33, 71] b	41 [34, 68] b	30 [22, 37] c	<0.001
γ-GTP (U/l)	24 [17, 39] a	45 [30, 104] b	46 [32, 71] b	46 [32, 60] b	<0.001
Albumin (g/dl)	4.5 [4.3, 4.7] a	4.4 [4.2, 4.7] a	4.3 [4.2, 4.4] b	3.9 [3.5, 4.2] c	<0.001
TB (mg/dl)	0.90 [0.80, 1.10] a	0.78 [0.62, 0.95] a	0.85 [0.66, 1.01] a	1.06 [0.69, 1.86] a	0.085
FBS (mg/dl)	96 [93, 102] a	100 [95, 113] ab	107 [102, 134] bc	123 [101, 140] c	<0.001
HbA1c (%)	5.60 [5.38, 5.80] a	6.0 [5.6, 6.2] b	6.3 [5.9, 6.9] c	6.6 [6.10, 6.90] c	<0.001
FIB4 index	1.05 [0.72, 1.29] a	0.91 [0.66, 1.25] a	2.12 [1.57, 2.90] b	4.18 [3.13, 5.44] c	<0.001
NFS	-2.35 [-2.94, -1.94] a	-2.85 [-3.33, -2.01] a	-0.48 [-0.86, 0.34] b	1.49 [1.17, 2.11] c	<0.001

NAFLD, nonalcoholic fatty liver disease; DM, diabetes mellitus; HTN, hypertension; HL, hyperlipidemia; sBP, systolic blood pressure; dBP, diastolic blood pressure; BMI, body mass index; AST, aspartate aminotransferase; ALT, alanine aminotransferase; γ-GTP, γ-glutamyl transpeptidase; TB, Total bilirubin; FBS, fasting blood sugar; NFS, NAFLD fibrosis score. Continuous variables are mean ± standard deviation or median (interquartile range [Q1, Q3]). There are significant differences if different alphabets are labeled (i.e. a vs. b).
